# Monitoring COVID-19 related changes in population mental health: Carmen Rodríguez-Blázquez

**DOI:** 10.1093/eurpub/ckac129.276

**Published:** 2022-10-25

**Authors:** C Rodríguez-Blázquez, S Aldridge, E Bernal-Delgado, L Dolanski-Aghamanoukjan, F Estupiñán-Romero, C Garriga, M Gissler, RA Lyons, S Sagerschnig, H Tolonen

**Affiliations:** 1 Instituto de Salud Carlos III, Madrid, Spain; 2 Population Data Science, Swansea University Medical School, Swansea, UK; 3 Institute for Health Sciences in Aragon, Zaragoza, Spain; 4 Austrian National Public Health Institute, Vienna, Austria; 5 Finnish Institute for Health and Welfare, Helsinki, Finland; 6 Karolinska Institutet, Stockholm, Sweden

## Abstract

**Background:**

The COVID-19 pandemic, and its consequences in terms of control measures and restrictions to normal life, has affected the population mental health. One of the four case studies from the Population Health Information Research Infrastructure (PHIRI) for COVID-19 is focused on mental health with the objective to measure changes in incidence of mental health problems associated with the COVID-19 pandemic in several European countries.

**Methods:**

Using electronic health records (EHR), data on new episodes of depression or anxiety, prescription of antidepressants and anxiolytics, and visits to primary care, specialized care or emergency units with an episode of depression/anxiety, were collected by participant data hubs at national/regional level for the period 2017-2021. A common data model to collect the data was defined for all participating data hubs and analysis of status prior and during the COVID-19 pandemic was performed using R.

**Results:**

Data hubs from Austria, Finland, Spain (Aragon), and United Kingdom (Wales) were able to provide aggregated results from raw individual-level data. Preliminary analysis of trends suggests a decrease in new cases of depression and anxiety in the pandemic period (2020-2021) in comparison with previous years. Different trends were observed between data hubs regarding prescription of drugs and the number of primary/specialized care visits due to depression or anxiety. Issues in the access to data in some of the participating data hubs were observed, related to ethical and legal matters, and the lack of centralized registers and of private consultations statistics.

**Conclusions:**

The results of this use case show that EHR for the secondary use can be retrieved in a common way across Europe to analyse and compare the impact of COVID-19 in population mental health in European countries. However, the process is more complicated and time consuming than expected.

